# Online programs to strengthen the mental health of medical students: A systematic review of the literature

**DOI:** 10.1080/10872981.2022.2082909

**Published:** 2022-06-01

**Authors:** Patrizia Ungar, Ann-Kathrin Schindler, Sabine Polujanski, Thomas Rotthoff

**Affiliations:** Department of Medical Education (DEMEDA), University of Augsburg, Augsburg, Germany

**Keywords:** Medical students, mental health, promotion, prevention, online programs

## Abstract

Medical students have been shown to be vulnerable to mental stress. Strengthening individual protective characteristics can be one cornerstone for promoting medical students’ mental health and thereby preventing mental disorders. Online programs are an opportunity to provide appropriate options that have the advantage of being accessible from anywhere, at any time, and with a low entry threshold. This review provides a literature overview of current online programs for medical students. The findings can serve as a point of reference for designing effective online programs for mental health-promotion and mental disorder-prevention in medical curricula. We applied a systematic literature search in PubMed, ERIC, Cochrane, and Web of Science. Programs offered had to be web-based, and the addressed group had to be medical students. Protective individual characteristics for mental health and information on the programs’ effectiveness were included in the search. As outcomes, we included mental health, burnout, symptoms of depression, anxiety, and well-being. The search yielded 723 articles; of them, 11 met the inclusion criteria. Programs found were grouped according to their focus: mental health literacy, mindfulness, based on Cognitive Behavioral Therapy, or peer support. Two studies showed significant reductions in perceived stress; one study indicated reduced burnout levels. One program had significant immediate effects on mindfulness, empathy, and resilience; two studies indicated strengthening coping strategies. Two programs were qualitatively assessed as helpful; two studies are ongoing. Nine studies lacked control groups; two randomized controlled trials were ongoing. Only a few online programs with limited evidence of effectiveness were found. They addressed protective individual characteristics, highlighting their importance for mental health. Thus, more health-promoting and mental disorder-preventing programs with high-quality effectiveness studies are necessary. An integration of such programs into curricula would allow for greater utilization and could give greater emphasis to and prioritize mental health in medical education.

## Introduction

University education is a special and challenging period of life that, while offering many new experiences, can be associated with stress and psychological strain [1,2]. University students worldwide experience a decrease in well-being and an increase in mental morbidity [3,4]. The reasons described are unfamiliarity as well as new situations [5], theses, exams, and high demands on oneself [6]. The World Health Organization (WHO) has addressed this issue through the WHO World Mental Health International College Student (WMH-ICS) initiative [4,7]. The goal of this initiative was to obtain longitudinal information about the prevalence and impact of mental health disorders among university students worldwide, as well as to lay the groundwork for the implementation of preventive interventions [2]. Results showed that 20.3% of university students were affected by mental disorders such as major depression [4,8], which lead to problems with home management, college-related work, close relationships, and social life [2]. Moreover, the study identified a low utilization of related treatments [4,8] among a large proportion of university students.

## Trends in mental health among medical students

Although not separately analyzed in the representative WMH-ICS study, there is a robust body of studies that identified medical students, in particular, as vulnerable to worrying mental health developments [9–15]. In a meta-analysis of 167 cross-sectional studies (*N* = 116,628) and 16 longitudinal studies from 43 countries, Rotenstein et al. reported an estimated overall prevalence of depressive symptoms of 27.2% (range: 9.3%–55.9%) [13,14]. Moreover, medical students show significantly higher rates of anxiety symptoms compared to the general population [15], and report fatigue, nervousness, uneasiness, lack of sleep, and feeling tired all the time [15]. Described reasons especially for medical students, are the amount of learning materials, perfectionism tendencies, and, in clinical phases, death and critically ill patients [10,14].

In accordance with the described trends, medical students should be preventively assisted in maintaining their mental health as early as possible to prevent the development of mental disorders [16]. Reforms in medical education have reacted to these findings and claim a stronger focus on conceptualizing curricula considering mental health [17]. As it is required that students should be seen as individuals *within* their educational context [17], *curricular* as well as *individual* interventions play a significant role in the conceptualization of curricula that consider students’ mental health. Curricular interventions subsume, for example, changes to course content [17,18], reduction of workloads, or straightened timetables. Individual interventions focus on individual characteristics [18,19] and can be thought of as the promotion of mental health, prevention of mental disorders, or treatment of mental disorders [20]. *Promotion* aims to strengthen positive aspects of mental health – such as characteristics that have been shown in the literature to be protective of mental health [20]; *prevention* aims to reduce the likelihood of developing mental disorders in the general population or in individuals who are identified as being at risk of a future disorder, and *treatment* is provided to people with diagnosed mental disorders [20]. Slavin [14] stated that promotion and prevention over treatment is a key point in improving medical students’ mental health.

## Relevance of online programs to promote mental health and prevent mental disorders

The described low uptake of personal consultation for mental health support by medical students [14,21] may be explained by fears of stigmatization, shame, barriers to time and place, and distrust of support offers [1,9,22–26]. New technologies have facilitated online mental health support that may overcome some of these barriers as they can be accessed anonymously from anywhere and at any time and can be used individually in a safe or well-known setting [1,9,27–29]. Therefore, online programs can be a cornerstone in creating medical studies that support students’ mental health.

In our overall project aim to design an online program to promote medical students’ mental health and thereby prevent the development of mental disorders, we identified a lack of overview on existing programs that are specifically designed for medical students. To address this research gap, we conducted a systematic search of the literature. We aimed to identify existing online programs designed for medical students’ mental health promotion and mental disorder prevention to give an overview on protective characteristics they address as well as the measures employed to assess their effectiveness. This systematic review of the literature can serve as an orientation for medical schools in conceptualizing low-threshold online services for mental health promotion and prevention.

As mental health promotion and individual prevention can be implemented by strengthening protective individual characteristics [20], we now focus on these characteristics.

## Protective individual characteristics

To define our search term, we screened the evidence on individual characteristics that were identified to be protective for mental health as they promote well-being and/or reduce the risk of mental disorders. Therefore, their addressing in online programs can be considered relevant:

### Self-efficacy

Self-efficacy is the conviction that one can influence and overcome challenges and difficult situations through one’s own strength and actions [30]. Self-efficacy has been shown to be predictive of feelings of stress in medical situations characterized by uncertainty [31] and to be trainable in health professional education [32].

### Resilience

Resilience describes a person’s ability to respond flexibly and appropriately to demands in changing situations and to cope with or recover from difficult or stressful situations without suffering any consequential psychological damage [33]. Like self-efficacy, resilience has been shown to be a modifiable construct [34,35].

### Empathy

Empathy describes a person’s ability to recognize and feel the feelings of others [36,37]. Lack of empathy has been shown to negatively correlate with well-being [37,38] and positively correlate with the development of burnout [39–42]. A recent German study involving medical students indicated that empathy can be strengthened through empathy training with standardized patients [43]. These findings suggest that students’ empathy can be promoted by appropriate measures.

### Coping and self-regulation strategies

Strategies that a person uses to cope with stressful situations and experiences are generally summarized under the term ‘coping strategies’ [44]. Different coping strategies are used by medical students and physicians, as shown by Lemaire and Wallace [45]. Some of these strategies (e.g., self-regulation strategies) correlate negatively with emotional exhaustion as a symptom of burnout and have been shown to help in coping with challenging situations in the medical profession [45]. Various aspects of self-regulation, which are frequently distinguished and mentioned as relevant subfacets [46], such as cognitive and metacognitive self-regulation, academic self-control, motivation, and emotional regulation, correlate negatively with the development of burnout [47,48].

### Mindfulness

Mindfulness is defined as the quality of awareness that occurs when intentionally focusing on experiencing the present moment in an accepting and nonjudgmental manner [49]. It has become an increasingly prominent psychological construct addressed in interventions worldwide [50]. Mindfulness-based interventions have been shown to reduce depression among medical and premedical students [51].

### Self-compassion

Self-compassion entails self-kindness, a sense of common humanity, and a balanced, mindful relationship with unpleasant thoughts and emotions [52–54]. Self-compassion has been shown to be associated with fewer depressive symptoms and burnout [53,55,56].

## Materials and methods

We conducted a systematic review of the literature using the Preferred Reporting Items for Systematic Reviews and Meta-Analyses (PRISMA) checklist as a guide [57].

### Information sources

We assessed the PubMed, Cochrane, Web of Science, and ERIC databases. The search terms had to be included either in the title or abstract (PubMed and ERIC) or in the title, abstract, or as a keyword (Cochrane, and Web of Science), as these served as the basis for screening the findings.

### Search strategy

Based on the first peer review of this article, we extended the search string and conducted the final search post review in January 2022. The search term definition was guided by the following premises:

1. The target group had to be medical students. Various denominations were included.
2. As we focused on online programs, the search strings had to include the word ‘online’ and suitable alternatives.
3. Studies had to be about an intervention or program with a preventive character.
4. The promotion of mental health and well-being and the prevention of depression, burnout, or anxiety had to be the aim of the online program.
5. The online program had to address at least one of the described protective individual characteristics.

In the search term, premises 1–5 were connected by an AND; alternatives in wording and spelling were connected within each premise by an OR:

((medical students OR students of medicine OR medicine students OR medical education)

AND

(online OR web-based OR webbased OR web based OR internet-based OR internetbased OR internet based OR digital OR telehealth OR telemedicine)

AND

(intervention OR prevention OR training OR programme OR program OR offering OR offer)

AND

(mental health OR depression OR depressive symptoms OR depressivity OR burn-out OR burnout OR burn out OR well-being OR wellbeing OR anxiety)

AND

(self-efficacy OR selfefficacy OR self efficacy OR resilience OR empathy OR coping strategies OR self-regulation OR selfregulation OR self regulation OR mindfulness OR self-compassion OR selfcompassion OR self compassion))

We considered articles starting in 2000 given the study topic of online, web-based, or digital programs.

### Selection and data collection process

Duplicated papers were excluded. Studies were screened for inclusion in the following way: One reviewer went over all the abstracts to remove the articles that certainly did not meet the inclusion criteria in accordance with premises 1–5.

The full text of all the remaining articles was reviewed independently by two reviewers. The results were discussed and discrepancies investigated until a consensus was reached. The remaining articles that formed the corpus for the results were subjected to the synthesis method. If the full text of an article could not be accessed, the article’s authors were contacted directly.

### Eligibility criteria

Articles were included if they fulfilled the following eligibility criteria:

1. The study reports an empirical study;
2. The study was conducted in one or more universities;
3. The article provided any information on an accompanying research procedure that investigated the impact or effects of the intervention or program (both quantitative and qualitative assessments were accepted); and
4. The article was published in an English- or German-language scientific journal.

### Study quality and risk of bias assessment

To estimate the risk of bias, each study’s methodological quality was assessed based on the criteria established by Jadad et al. [58]. It is important to state here that, despite the possible risk of bias of the included studies being discussed by the two reviewers, a possible risk of bias in one study did not form a criterion for this study to be excluded from our review.

### Synthesis method

We inductively identified categories to (1) group the identified online programs in accordance with their overall approach. (2) Within these, we sorted the studies by their provided evidence in the following order: Randomized controlled trials (RCTs) were presented above studies without a control group. Studies with a higher number of participants were presented above studies with a lower number of participants. Significant results were presented above results without significance. If there were two studies on the same or nearly the same intervention, they were reported chronologically.

## Results

### Selected studies

**Figure 1.**
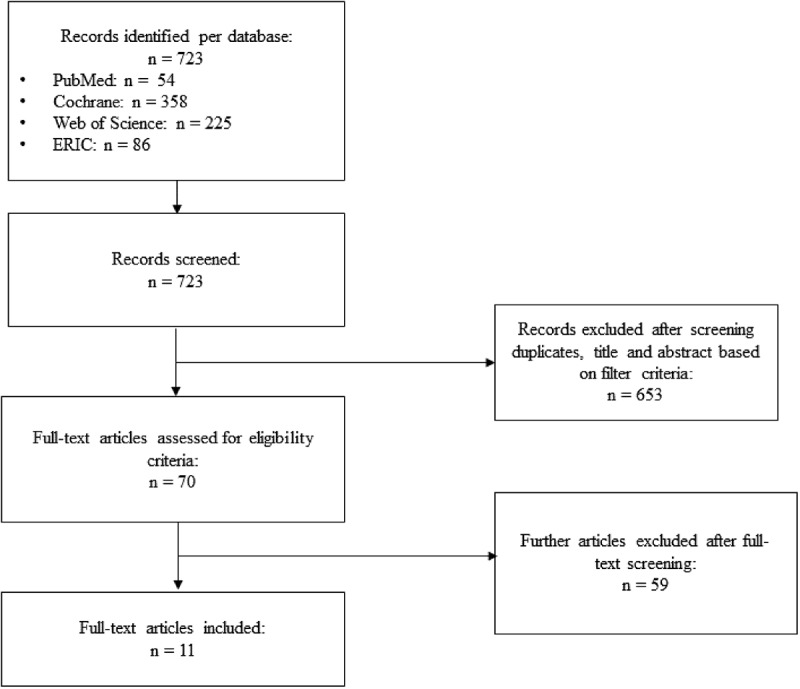
Flowchart for selecting the search results.

As illustrated in Figure 1, 723 records were found in the four databases using the search string described above. In accordance with this selection procedure, 70 studies were retrieved for detailed examination, which resulted in 11 articles that were ultimately included in the systematic review.

### Study characteristics

Five studies were conducted in the USA. One study was conducted in each of the following countries: Canada, Finland, Spain, Chile, Australia, and Iran. There were two cases in which the same group of authors conducted two studies in each case. Two studies were explicitly named RCTs, whereas most studies were conducted with a pre-posttest design without a control group.

### Study outcomes

For the 11 studies (see Table 1), we identified four approaches that serve as the logic for the presentation of the results: mental health literacy, mindfulness/meditation, based on internet-based Cognitive–Behavioral Therapy, and peer support.

**Table 1.** Details of the studies included in the review

Approach	Authors, year, country	Objectives	Content of the intervention	Didactic format	Provided evidence	Participants	Key instruments/measurements	Key results
Mental health literacy (MHL)	Kurki et al (2021), Finland [60]	Assessment of a MHL program that covered blended life skills and mindfulness activities	Intervention comprised the following 3 themes: 1) Skills for independent life, study skills, relationships; 2) Stress management and mental health sustaining strategies; 3) Information about mental disorders, treatment, and help seeking Additional mindfulness component: theory of mindfulness and exercises based on mindfulness-based stress reduction	Two 60-minute lectures, four weeks apart, and online self-learning material in between; links to information sources and videos; mindfulness component was provided with a series of audio tapes	One group, quasi-experimental pretest-posttest design, 2-month follow-up evaluation	N = 374 first-year medical students, of which 158 completed all stages	Mental Health Knowledge questionnaire, Stigma questionnaire, The Help-seeking questionnaire, The General Health Questionnaire, PSS, Client Satisfaction Questionnaire (CSQ-1)	Scores for mental health knowledge improved and emotional symptoms were alleviated at posttest and 2-month follow-up; stress levels reduced and attitudes towards help-seeking improved after the program, but these changes were not maintained at 2-month follow-up
Mindfulness/meditation	Villalon Lopez (2021), Chile [62]	Assessment of the effectiveness of a brief online mindfulness, compassion and intercare based intervention (MIIM)	Mindfulness-based interventions such as mindfulness based cognitive training, self-compassion and cognitive based compassion training; modules consisting in meditation practices, inquiry and self or group reflections on topics such as mindfulness, automatic pilot, mind wandering, acceptance, gratitude and compassion, care resources and intercare	1 hour per week for 4 weeks, 4 synchronic ZOOM group sessions and home practice	Registered RCT; measurements at the beginning, 1-month and 3-month follow-up	Current enrollment: N = 360 medical students	PHQ9, GAD-7, Mental Health Continuum Scale – Short Form (MHC-14), FFMQ-SF	No results reported yet
	Rojas (2020), Spain [63]	Evaluation of the efficacy and mechanisms of change of Compassion Cultivation Training (CTT)	Online version of the CCT: behavioral meditation program designed at Stanford University comprising six sequential steps: 1) Settling the mind; 2) Loving-kindness and compassion for a loved one; 3) Self-directed loving-kindness and compassion; 4) Common humanity; 5) Cultivating compassion for others; 6) Active compassion	8-week program; weekly 2-hour online sessions, daily 20–30-minute online meditation and compassion instructions, guided by a certified instructor from the Compassion Institute	Registered RCT; 2 groups: intervention condition and waitlist control condition; measurements at pre-, inter-, and post-intervention, as well as 2-month and 6-month follow-up; program adherence and fidelity will be monitored through revisions of the recorded sessions	Actual enrollment: N = 40 medical students	Primary Outcome Measures: Compassion Scale Pommier (CSP), Self-Compassion Scale (SCS-SF), Interpersonal Reactivity Index (IRI) for empathy, 21-item Depression Anxiety Stress Scale (DASS-21), Pemperton Happiness Index (PHI) Secondary Outcome Measures: FFMQ, MBI, BRS, Difficulties in Emotion Regulations Scale (DERS), single-item to measure daily formal meditation practices and informal compassion practices, single-item to measure state changes in compassion, mindfulness, psychological distress, well-being, and cognitive-emotional regulation processes	No results reported yet
	Kemper & Khirallah (2015), USA [64]	Measurement of acute effects of online mind-body skills training (MBS) on stress, resilience, mindfulness, and empathy	Topics of meditation: focused attention meditation (relaxation response), mindfulness meditation, positive affect meditation, guided imagery/hypnosis; at least one module had to be completed which participants could choose freely	12 modules, divided into 4 general topics;1-hour modules with online lessons for meditation practices	Prospective cohort study, measurements pre- and immediately post-training; no control group; here, acute effects were measured as a part of a larger evaluation (mentioned below)	N = 513 enrollees including dietitians, nurses, physicians, social workers, clinical trainees, and health researchers; about 1/4 were clinical trainees	Data from the 5 modules that had at least 100 enrollees within the study period were analyzed; Visual analog scale (VAS) for stress, relaxation and resilience; 10-item Perceived Stress Scale (PSS), 10-item Cognitive and Affective Mindfulness Scale – Revised (CAMS-R), Mindful Attention Awareness Scale (MAAS), Brief Resilience Scale (BRS), IRI (Empathic Concern (EC) and Perspective Taking (PT) subscales), open-ended questions	Significant improvements in stress, mindfulness, empathy and resilience
	Kemper et al (2015), USA [65]	Investigation of the longer-term impact of online mind-body skills training on stress, mindfulness, resilience, empathy, self-compassion	MBS as mentioned above; this time, participants had to have finished the whole intervention	1-hour modules with online lessons for meditation practices	Cohort trial, pre- and post-training survey after 12 weeks, no control group	N = 103 participants who had finished the whole intervention	10-item CAMS-R, SCS-SF, 10-item Calm, Compassionate Care Scale (CCCS), 6-item BRS, 7-item Empathic Concern Scale (ECS) (IRI subscale), 7-item PT scale (IRI subscale), 5-item Santa Clara Brief Compassion Scale (SCBCS), module engagement	Significant improvements over 12 weeks in stress, mindfulness, self-compassion, and confidence in providing calm, compassionate care; no significant improvements in empathy and resilience
	Moore et al (2020), Australia [66]	Investigation of the effectiveness of an online mindfulness training for self-compassion, compassion levels, and stress	Mindfulness training providing information about mindfulness, regular mindfulness and meditation techniques and short sessions for motivation and to answer questions	8-week training; 10-minute weekly mini-lectures, 5-minute daily guided mindfulness meditation sessions, online video conference sessions	Prospective mixed method cohort study, no control group	N = 47 medical students	PSS, SCS, Compassion Scale (CS)	Statistically significant reduction in participants’ perceived stress levels and a significant increase in self-compassion at 4-month follow-up
	Danilewitz et al (2018), Canada [67]	To investigate the efficacy and feasibility of an online mindfulness-based program on burnout, empathy, mindfulness, self-compassion	MIND-MED: online mindfulness intervention; techniques like body scan, meditations, mindfulness eating, mindfulness yoga, mindfulness in daily activities	7 modules, 25–35 minutes each; audio files, video instructions, text and reading material, links to relevant other websites; weekly reminder E-mails	Prospective pilot cohort study, pre- and post-assessments, no control group	N = 52 medical students (convenience sample), of which 45 finished at least one module	Maslach Burnout Inventory (MBI), Jefferson Scale of Empathy – medical student version (JSE-M), Five Face of Mindfulness Questionnaire – short form (FFMQ-SF), Self-Compassion Scale – short form (SCS-SF), Interpersonal Reactivity Index (IRI) for empathy	No changes in burnout and empathy, high acceptance
Based on internet-based Cognitive Behavioral Therapy (iCBT)	Lattie et al (2017), USA [68]	To assess acceptability and usability of an iCBT program, and to examine the impact of the program on perceived stress, feelings of burnout, quality of life, and the development of cognitive and behavioral coping strategies	Program called ThinkFeelDo; topics such as behavioral activation, cognitive restructuring, and managing anxiety	6-week program; mobile accessible web program, 14 10-minute online lessons with text and video and 5 interactive tools including activity monitoring, cognitive restructuring, goal setting, relaxation, and mood tracking	Pre- and postintervention assessments, no control group	N = 14 medical students	Frequency and duration of site use, usability and user feedback with the Usefulness, Satisfaction, and Ease of use (USE) questionnaire; 9-item semi-structured interview via telephone; PSS, Medical Student Well-Being Index (MSWBI), Cognitive and Behavioral Response to Stress Scale (CB-RSS) to measure frequency and usefulness of cognitive and behavioral coping skills	High acceptability and usability; at the end of the program, fewer participants reported feeling burned out from medical school compared to baseline
	Lattie et al (2019), USA [69]	Primary aims: to examine program usage and the demographics of students who used the program. Secondary aims: to examine the impact of the program on perceived stress, quality of life, and the development of cognitive and behavioral coping skills	Slightly modified version of the ThinkFeelDo-program above	16-week program with 16 10-minute online lessons and again 5 interactive tools; content was the same as mentioned above	Pre- and postintervention assessments, no control group	N = 53 first year medical students	Frequency and duration of site use; MSWBI, CB-RSS, Patient Health Questionnaire-8 (PHQ-8), Generalized Anxiety Disorder Scale-7 (GAD-7)	No significant changes in PHQ-8, GAD-7 or PSS; slight increase in the frequency of cognitive coping strategy use; interest in this online program was high, actual program usage was low
Peer support	George et al (2013), USA [70]	To investigate the effect of peer-support via Facebook-based stress management group for first-year medical students	Stress management during the first eleven weeks of medical school; peer-support and assistance for building coping strategies	Facebook group, daily posts with videos or text, online peer-to-peer communication	Qualitative analysis of open questions (post-treatment), no control group	N = 95 first year medical students	Open questions, qualitative measurements	High engagement, better stress management and coping strategies afterwards
	Kazerooni et al (2020), Iran [72]	Investigation of the effectiveness of a Social-Media-platform for support of junior medical students during Covid-19 pandemic	Peer-support to maintain online social contacts and to offer mutual assistance	Social-Media-platform, online peer-to-peer communication	Post-intervention questionnaire measures, no control group	N = 371 junior medical students, 10 senior medical students	Valid and reliable questionnaire was used to measure the effect of this activity; not further specified	71% of junior medical students believed the platform had a significant impact on helping them adjust faster to these emergency conditions, students generally reported that they had benefited from the intervention

### Mental health literacy

We found one study on a mental health literacy (MHL) intervention that was implemented in 2020 during the COVID-19 pandemic at the University of Turku, Finland. MHL is a component of health literacy that aims to increase knowledge about mental health and positive attitudes toward available mental health services [59,60]. Insufficient MHL may result in increased stigma, lack of recognition of mental health problems, and barriers to help seeking, which is associated with increased stress [60]. Therefore, the aim of this intervention was to reduce stress and thereby enhance well-being by increasing mental health awareness and reducing the stigma associated with mental health problems. This intervention was called Transition program and based on the Canadian Transitions program, which is a program in the form of a booklet (available in hard copy, ebook, and mobile app formats [61]) that combines MHL with comprehensive life-skills resources to support students in their transition to university. The program here was offered completely digitally and consisted of two lectures and self-learning digital materials that focus on three themes. Theme 1 comprised skills for independent life, study skills, and relationships. Theme 2 addressed stress management skills and strategies to sustain mental health. In theme 3, users were provided with information about the variety of mental disorders, treatment, and help seeking [60]. Additionally, the Transition program included a mindfulness component with a series of audio tapes on the theory and exercises to learn about mindfulness as a stress-reduction strategy. As mindfulness was not the focus, we grouped the Transition program distinctly from approach 2. The students’ mental health knowledge, their attitudes toward help-seeking, and their emotional wellbeing improved significantly after the program, and they showed significantly reduced stress levels. Changes concerning mental health knowledge and emotional well-being remained at follow-up, whereas changes in stress levels and help-seeking attitudes were not maintained two months after the program.

### Mindfulness/meditation

The only two RCT studies found in this systematic review are still ongoing. The documents found are registered study protocols.

One registered (August 2021) mindfulness intervention was offered at the University Diego Portales in Santiago de Chile during the COVID-19 pandemic [62]. The aim of the study is to evaluate the effectiveness of a brief online mindfulness, compassion, and intercare-based intervention to prevent adverse impacts of the pandemic on the mental health of medical students. For that purpose, the protocol reported an RCT in which 360 participants were randomly assigned to 1) a mindfulness, compassion, and intercare-based intervention, 2) a psychoeducational intervention, or 3) a waiting control group. The mindfulness intervention consisted of mindfulness-based cognitive training and teaching techniques for practicing at home. The psychoeducational intervention included psychoeducation about stress, anxiety, self-care, and effective communication. In addition, it was reported that all participants were offered psychological counseling if they showed a high score on depression or anxiety symptoms. All participants had academic breaks of two weeks per semester and flexibility in submitting work, attending practical activities, and taking exams [62]. The results have not been published yet.

A second RCT registration protocol (December 2020) called Compassion Cultivation Training (CTT) was conducted at Stanford University, USA. An 8-week mediation program was offered as an online version with weekly online sessions, supplemented with daily exercises led by a certified instructor to support medical students’ mental health during the COVID-19 pandemic. The objective was to determine the effectiveness of this training on self-compassion, empathy, resilience, mindfulness, and emotional regulation, as well as on depression, anxiety, stress, and burnout [63]. N = 40 medical students are currently enrolled. Again, no results have yet been published.

Two articles by Kemper et al. reported on a program for healthcare professionals in general, including medical students, doctors, nurses, and social workers [64,65] at the Ohio State University College of Medicine. The online mind–body skills program (MBS) comprised 12 1-h modules that were divided into four general topics: focused attention meditation (relaxation response), mindfulness meditation, positive affect meditation, and guided imagery/hypnosis [64,65]. The acute effects of this program on mindfulness, stress, empathy, and resilience in 513 participants were investigated. No control group was reported. The participants had to complete at least one module of the program and were free to choose the module(s) they wanted to work on. Through self-assessment scales, significant improvements in mindfulness, empathy, and resilience, as well as significantly lower levels of perceived stress, were noted [64]. Self-assessment scales were used again in the second study. 12 weeks after the MBS (103 participants who completed all modules of the training), significant effects could be shown for perceived stress, mindfulness, and self-compassion, but not for empathy and resilience [65].

A pilot study by Moore et al. conducted at the Australian Rural Clinical School reported on the effects of an 8-week mindfulness program for medical students on self-compassion and stress [66]. The program included short online lectures on mindfulness, guided mindfulness meditation sessions, and videoconference sessions for support and motivation. Lectures included topics such as stress reduction and performance, reducing distraction and procrastination, and mindful use of technology. Mindfulness sessions included, for example, techniques such as body scans, mindful breathing, and mindful listening. Moore et al. conducted a single-arm prospective mixed-method cohort study that included 47 medical students and no control group. Half of the participants used the program at least once a week. At the follow-up survey 4 months after program completion, the researchers found a significant increase in the students’ self-compassion and a significant reduction in perceived stress.

Danilewitz et al. developed and evaluated a web-based mindfulness program called MIND-MED to increase empathy, mindfulness, and self-compassion and to reduce burnout in medical students at the University of Ottawa, Canada [67]. MIND-MED consists of seven sequential online modules that are accessible to students for purely self-administered use over one semester. It suggests techniques such as body scans, meditations, mindful eating exercises, and yoga. Additionally, it provides tips for promoting mindfulness in everyday activities. Furthermore, relevant reading material about mindfulness and student well-being, as well as links to other websites that contained information about mindfulness, were provided. The researchers offered MIND-MED as a voluntary additional program in a prospective pilot to 45 medical students without a control group. The program had high acceptance but no significant effects on empathy and burnout. Some students stated that they did not find enough time or were too stressed to regularly perform the meditation exercises [67].

### Based on internet-based cognitive-behavioral therapy

Lattie et al. [68] developed ThinkFeelDo, a 6-week online program based on internet-based Cognitive-Behavioral Therapy consisting of online lessons and interactive online tools to promote cognitive and behavioral coping strategies and to reduce stress and burnout among first-year medical students. Through 10-minute online lessons, participants from the University of Illinois at Chicago learned about the topics of behavioral activation, cognitive restructuring, and dealing with anxiety by using text and videos [68]. In addition, an associated website offers interactive tools, such as activity monitoring, mood tracking, documentation of cognitive restructuring and goal setting, and a relaxation exercise to implement the content learned in the lessons [68]. After the completion of ThinkFeelDo, participants showed high acceptance and usability scores, and an increase in cognitive and behavioral coping strategies as well as a decrease in burnout were measured in the pilot study [68].

Two years later, the authors investigated the effect of a slightly modified version of ThinkFeelDo on the well-being, stress, depressive symptoms, anxiety disorders, and coping strategies of first-year medical students [69]. This time, the program ran for 16 weeks. There were slight improvements in the medical students’ coping strategies, but no significant pre–post differences in depression and anxiety symptoms, which were initially low, were observed. ThinkFeelDo was rarely used repeatedly, although students reported having a high interest in it [69]. In both studies, the authors reported no control group.

### Peer support

George et al. [70] used Facebook to establish online support for first-year medical students during the initial 11 weeks at the Pennsylvania State Hershey College of Medicine. They created a Facebook stress management group that provided peer support based on a blend of CBT and Lazarus & Folkman’s model of stress and coping [71]. Every day, new content was posted that provided personal video narratives from older medical students, educational information, study tips, and stress management resources, such as problem-focused coping strategies (e.g., changing the environment by reframing or relaxing). In total, 95 medical students used the service and reported better stress management and coping strategies through open self-report items. No control group was included [70].

Kazerooni et al. reported on an offer at the Shiraz University of Medical Sciences, Iran, which was developed last year during the COVID-19 pandemic [72]. A social media platform was created for cross-semester peer exchange to reduce stress, anxiety, and psychological strain and to support the development of strategies to cope with this unfamiliar pandemic situation. It involved senior medical students assisting junior medical students in coping with anxiety and stress brought about by the COVID‐19 pandemic. In total, 371 junior students tested the platform as mentees, sharing psychological difficulties, worries, and fears, and 10 senior students acted as coaches [72]. In their role as coaches, they recommended stress management and relaxation techniques, engagement in exercise, maintenance of online contact with family and friends, and time management during quarantine; moreover, they were generally available as contact persons. The majority (71%) of the junior students found peer support helpful in adapting to unfamiliar and stressful situations, and that it was a unique experience beneficial for their professional growth [72]. Concrete measures of stress, anxiety, or other constructs, as well as coping strategies, were not reported, nor was a control group installed. Because of the COVID-19 pandemic, the initial intervention was conducted virtually via the social media platform for about one year; however, it has since continued in hybrid form and is now both in person and virtual. Content is created and posted online, and mentees are mentored through private (& peer) meetings – both in person and virtually (A. R. Kazerooni, personal communication, March 1^st^, 2022).

## Discussion

With this systematic review of the literature, we aimed to identify online programs developed for mental health promotion and mental disorder prevention in medical students. Applying a systematic search, we identified 11 studies on nine different online programs – two programs were evaluated in two consecutive studies – which addressed medical students’ mental health. The studies included one MHL intervention [60], an internet-based program based on CBT [68,69], MBS training [64,65] as well as further meditation and mindfulness programs [62,63,66,67]. Two programs used the supportive effects of peer support [70,72]. Didactical formats differed and included online lectures, guided lessons, or sessions with practice exercises, interactive tools, and online peer-to-peer communication via social media platforms.

Overall, the accompanying studies on the effects or effectiveness of each program were methodologically of rather low quality and significance, but the reported results of their overall good acceptance suggest that such programs are quite well received by their users. The only two RCTs we found are still ongoing. The small number of programs found also indicates that, to date, there are few online programs especially designed for medical students.

The programs found addressed protective individual characteristics such as empathy, resilience, mindfulness, self-compassion, or coping strategies, and focused on outcomes such as perceived stress, depressive symptoms, or burnout.

Based on our systematic review of the literature, we derive the following implications:

1. Digital support seems to attract interest

The predominantly good acceptance of online programs indicates that medical students are interested in digital support to maintain or strengthen their mental health. In the current pandemic, the advantages of online programs, such as the possibility of using them independently of time and place, are particularly apparent.

(2) Definition of targeted learners

None of the included studies reported a specific needs assessment of the target population (i.e., medical students) before developing the program. With a longitudinal questionnaire monitoring mental health development during medical school, both stress hot spots and students in need can be identified [73]. In this way, a student-oriented, needs-based online program can be developed as a component of a curriculum that considers mental health.

(3) Need for effectiveness measures

Effectiveness measures were reported in most studies, but the samples were partly small and lacked a control group. Most included studies mentioned the need for further methodologically high-quality studies to verify the effectiveness of their measures [66–68,72].

In agreement with them, we also emphasize the need for high-quality studies on this topic to achieve more reliable and valid statements on its (differential) effectiveness—that is, to determine which individuals may particularly benefit from online programs and how to adapt the content of the program, if necessary.

(4) Mindfulness training and peer support showed a positive impact

Although the results of some of the studies need to be interpreted cautiously, they are nevertheless an initial indication of the positive impact of the approaches and content offered in the online programs on mental health. They indicated that short online sessions on relaxation and meditation and mindfulness training in general can strengthen protective characteristics, such as resilience and empathy, and therefore promote well-being as well as prevent the development of depression and burnout symptoms. Peer support was well accepted and considered helpful in adapting to stressful situations. As these two components had positive effects, future online programs should combine short online sessions on mindfulness training with peer support. Peer-to-peer communication should also be fostered to promote the exchange of experience and facilitate mutual support.

(5) Online programs should be an integrated component

The identified programs were offered to medical students as additional services which—as found in the study by Lattie et al. [69]—can risk continuous use because of time constraints or perception as additional effort.

These findings indicate a need for more programs that are wisely implemented into the curriculum as an integrated component. In this way, an add-on character and additional workload can be reduced, thereby achieving a sustainable combination of individual and structural interventions to promote mental health and prevent mental disorders in future physicians—in accordance with Slavin’s [17] understanding of students as individuals within their educational context. This finding could be useful to curriculum developers who plan medical students’ education within a curriculum that considers mental health: effective mental health promotion as well as effective prevention of mental disorders consist of both individual and structural interventions. This requires individual interventions to be embedded into a structurally (e.g., in terms of time schedule) adapted curriculum, as they are only one component in an entire setting; this means that structural changes in the curriculum itself, such as changes in scheduling or course content, are necessary.

(6) Dissemination of our findings

We sought programs specifically designed for medical students. Nevertheless, the findings of our review can be disseminated to other student groups as well as even other institutions in general, as the need for support to keep students and other community members (for example faculty, residents, and practitioners across health professions) mentally healthy is universal [16]. Tailored (i.e., developed on the basis of the needs of the respective target group) online programs as individual interventions that are structurally embedded into a curriculum can be a good way to promote mental health of other community members and in other institutions as well.

## Limitations

This review has some limitations. The only two RCT studies are registered protocols only; so far, they are without published results. Since all studies described the experience of only one institution, the generalizability of each of the findings is limited.

We inductively grouped the results into four approaches that appeared reasonable and most prominent based on the focus of the respective program. Nevertheless, the *mental health literacy* approach by Kurki et al. [60], included a mindfulness component with mindfulness elements as well. The potential effects of such additional components should be taken into account when interpreting the effectiveness of a program.

The approach *based on internet-based Cognitive Behavioral Therapy* is actually represented by only one intervention, which was studied twice – each time in a slightly modified form. Two studies grouped under *mindfulness/meditation* were conducted on the same intervention by the same group of authors, as reported. Thus, the sheer number of studies on these two approaches is not the same as the number of different interventions or programs.

Our review includes articles published in international journals and two registered trial protocols. Relevant literature may have been excluded, for example, from conference papers, conference proceedings, or theses, as well as in languages other than English or German.

Although the search string has been adjusted, there might be other individual protective characteristics that we did not include in our search but that might be relevant for mental health. Thus, some relevant online programs for medical students may have been overlooked.

## Conclusion

From this systematic review of the literature, which provides an initial overview of online programs to strengthen medical students’ mental health, we derive the following conclusions for mental health promotion and mental disorder-preventing programs in the university setting or, to go even further, in other institutions in general:

1. Online programs seem to be a good way to support individual mental health promotion and prevention by strengthening protective characteristics, also for target groups other than medical students.
2. To better understand the respective target groups and their needs, we suggest a precise needs assessment among them as a basis on which online programs are designed to ensure their necessity as well as their best possible fit.
3. Online programs would benefit from curricular integration. There is a need for a curricular – or more general – structural integration of such tailored online programs or interventions, for example by giving them a fixed and obligatory slot in the timetable so that students or other addressees have enough time and space to use and benefit from them. This again underlines the fact that individual and structural prevention should not be separated from each other but should be considered interacting components.
4. Effectiveness studies can help to further understand the contribution of online programs to mental health-promoting as well as mental disorder-preventing curricula.

## Abbreviations

BRS: Brief Resilience Scale
CAMS-R: Cognitive and Affective Mindfulness Scale – Revised
CB-RSS: Cognitive and Behavioral Response to Stress Scale
CBT: Cognitive Behavioral Therapy
CCCS: Calm, Compassionate Care Scale
CS: Compassion Scale
CSP: Compassion Scale Pommier
CSQ-1: Client Satisfaction Questionnaire
CTT: Compassion Cultivation Training
DASS: Depression Anxiety Stress Scale
DERS: Difficulties in Emotion Regulation Scale
ECS: Empathic Concern Scale
FFMQ: Five Facet of Mindfulness Questionnaire
FFMQ-SF: Five Facet of Mindfulness Questionnaire Short Form
GAD-7: Generalized Anxiety Disorder Scale 7
**IBAP**: Mindfuless based intercare program
iCBT: Internet-based Cognitive Behavioral Therapy
IRI: Interpersonal Reactivity Index
JSE-M: Jefferson Scale of Empathy – medical student version
MAAS: Mindful Attention Awareness Scale
MBI: Maslach Burnout Inventory
MBS: Mind-body skills training
MBCT: Mindfulness-based cognitive therapy
MBSR: Mindfulness-based stress reduction
MHL: Mental health literacy
MHC-14: Mental Health Continuum Scale – Short Form
MIIM: Mindfulness and Intercare Based Intervention for Medicine Students
MSWBI: Medical Student Well-Being Index
PHI: Pemperton Happiness Index
PHQ-9: Patient Health Questionnaire – 9
**PRISMA**: Preferred Reporting Items for Systematic Reviews and Meta-Analyses
PSS: Perceived Stress Scale
RCT: Randomized controlled trial
SCBCS: Santa Clara Brief Compassion Scale
SCS: Self-Compassion Scale
SCS-SF: Self-Compassion Scale Short Form – 12
USE: Usefulness, Satisfaction, and Ease of use
VAS: Visual analog scale
**WMH-ICS**: WHO World Mental Health International College Student initiative

## References

[1] Lutz-Kopp C, Meinhardt-Injac B, Luka-Krausgrill U. Psychische Belastung Studierender. Prävention und Gesundheitsförderung. 2019;14(3).

[2] Alonso J, Mortier P, Auerbach RP, et al. Severe role impairment associated with mental disorders: results of the WHO world mental health surveys international college student project. Depress Anxiety. 2018;35(9):802–15. 29847006 10.1002/da.22778 PMC6123270

[3] Andrews A, Chong JLY. Exploring the wellbeing of students studying at an Australian university. J Aust N Z Stud Serv Assoc. 2011;37:9–38.

[4] Auerbach RP, Alonso J, Axinn WG, et al. Mental disorders among college students in the world health organization world mental health surveys. Psychol Med. 2016;46(14):2955–2970. 27484622 10.1017/S0033291716001665 PMC5129654

[5] Conley CS, Kirsch AC, Dickson DA, et al. Negotiating the transition to college: developmental trajectories and gender differences in psychological functioning, cognitive-affective strategies, and social well-being. Emerging Adulthood. 2014;2(3):195–210.

[6] Herbst U, Voeth M, Eidhoff AT, et al. Studierendenstress in Deutschland. Eine empirische Untersuchung. Berlin: AOK-Bundesverband; 2016 cited 2021 Feb 28]. Available from 2021 Feb 28: https://www.ph-ludwigsburg.de/uploads/media/AOK_Studie_Stress.pdf.

[7] Kessler RC, Aguilar-Gaxiola S, Alonso J, et al. The WHO world mental health (WMH) surveys. Psychiatrie (Stuttgart). 2009;6(1):5–9. PMC2995950 21132091

[8] Auerbach RP, Mortier P, Bruffaerts R, et al. WHO world mental health surveys international college student project: prevalence and distribution of mental disorders. J Abnorm Psychol. 2018;127(7):623–638. 30211576 10.1037/abn0000362 PMC6193834

[9] Guille C, Zhao Z, Krystal J, et al. Web-Based cognitive behavioral therapy intervention for the prevention of suicidal ideation in medical interns: a randomized clinical trial. JAMA Psychiatry. 2015;72(12):1192–1198. 26535958 10.1001/jamapsychiatry.2015.1880 PMC4866804

[10] MacLean L, Booza J, Balon R. The impact of medical school on student mental health. Acad Psychiatry. 2016;40(1):89–91. 25749920 10.1007/s40596-015-0301-5

[11] Dahlin M, Joneborg N, Runeson B. Stress and depression among medical students: a cross-sectional study. Med Educ. 2005;39(6):594–604. 15910436 10.1111/j.1365-2929.2005.02176.x

[12] Dyrbye LN, Thomas MR, Massie FS, et al. Burnout and suicidal ideation among U.S. medical students. Ann Intern Med. 2008;149(5):334–341. 18765703 10.7326/0003-4819-149-5-200809020-00008

[13] Rotenstein LS, Ramos MA, Torre M, et al. Prevalence of depression, depressive symptoms, and suicidal ideation among medical students: a systematic review and meta-analysis. Jama. 2016;316(21):2214–2236. 27923088 10.1001/jama.2016.17324 PMC5613659

[14] Slavin SJ. Medical student mental health: culture, environment, and the need for change. Jama. 2016;316(21):2195–2196. 27923076 10.1001/jama.2016.16396

[15] Suarez DE, Cardozo AC, Ellmer D, et al. Short report: cross sectional comparison of anxiety and depression symptoms in medical students and the general population in Colombia. Psychol Health Med. 2021;26(3):375–380. 32314943 10.1080/13548506.2020.1757130

[16] Haramati A, Cotton S, Padmore JS, et al. Strategies to promote resilience, empathy and well-being in the health professions: insights from the 2015 CENTILE Conference. Med Teach. 2017;39(2):118–119. 28103729 10.1080/0142159X.2017.1279278

[17] Slavin SJ, Schindler DL, Chibnall JT. Medical student mental health 3.0: improving student wellness through curricular changes. Acad Med. 2014;89(4):573–577. 24556765 10.1097/ACM.0000000000000166 PMC4885556

[18] Schaefer C Gestärkt für den Lehrerberuf“: psychische Gesundheit durch Förderung berufsbezogener Kompetenzen; Entwicklung und Evaluation eines stärkenfokussierten Interventionsprogramms für Lehramtsstudierende “Strengthened for the teaching profession”: mental health by promoting professional skills; development and evaluation of a strengths-based intervention program for student teachers 2012.

[19] Awa WL, Plaumann M, Walter U. Burnout prevention: a review of intervention programs. Patient Educ Couns. 2010;78(2):184–190. 19467822 10.1016/j.pec.2009.04.008

[20] Purgato M, Uphoff E, Singh R, et al. Promotion, prevention and treatment interventions for mental health in low- and middle-income countries through a task-shifting approach. Epidemiol Psychiatr Sci. 2020;29:e150. 32744223 10.1017/S204579602000061X PMC7458538

[21] Coombes R. Medical students need better mental health support from universities, says BMA. Bmj. 2018;361:k2828. 29950519 10.1136/bmj.k2828

[22] Eisenberg D, Golberstein E, Gollust SE. Help-seeking and access to mental health care in a university student population. Med Care. 2007;45(7):594–601. 17571007 10.1097/MLR.0b013e31803bb4c1

[23] Eisenberg D, Hunt J, Speer N, et al. Mental health service utilization among college students in the USA. J Nerv Ment Dis. 2011;199(5):301–308. 21543948 10.1097/NMD.0b013e3182175123

[24] Mowbray CT, Megivern D, Mandiberg JM, et al. Campus mental health services: recommendations for change. Am J Orthopsychiatry. 2006;76(2):226–237. 16719642 10.1037/0002-9432.76.2.226

[25] Clement S, Schauman O, Graham T, et al. What is the impact of mental health-related stigma on help-seeking? A systematic review of quantitative and qualitative studies. Psychol Med. 2015;45(1):11–27. 24569086 10.1017/S0033291714000129

[26] Andrade LH, Alonso J, Mneimneh Z, et al. Barriers to mental health treatment: results from the WHO world mental health surveys. Psychol Med. 2014;44(6):1303–1317. 23931656 10.1017/S0033291713001943 PMC4100460

[27] Howells A, Ivtzan I, Eiroa-Orosa FJ. Putting the ‘app’ in happiness: a randomised controlled trial of a smartphone-based mindfulness intervention to enhance wellbeing. J Happiness Stud. 2016;17(1):163–185.

[28] Bush NE, Smolenski DJ, Denneson LM, et al. A virtual hope box: randomized controlled trial of a smartphone app for emotional regulation and coping with distress. Psychiatr Serv. 2017;68(4):330–336. 27842473 10.1176/appi.ps.201600283

[29] Patel V, Saxena S, Lund C, et al. The lancet commission on global mental health and sustainable development. Lancet. 2018;392(10157):1553–1598. 30314863 10.1016/S0140-6736(18)31612-X

[30] Stangl WS, Stangl W, Wien LF; 2021 cited 2022 Apr 16]. Available from 2022 Apr 16: https://lexikon.stangl.eu/1535/selbstwirksamkeit-selbstwirksamkeitserwartung.

[31] Huang I-C. Self-esteem, reaction to uncertainty, and physician practice variation: a study of resident physicians. Social Behav Personality. 1998;26(2):181–194.

[32] Jurkat H, Richter L, Cramer M, et al. Depressivität und Stressbewältigung bei Medizinstudierenden: eine Vergleichsuntersuchung des 1. und 7. Fachsemesters Humanmedizin. Nervenarzt. 2011;82(5). 10.1007/s00115-010-3039-z 21165590

[33] Stangl WR, Stangl W, Wien LF; 2021 cited 2021 Apr 16]. Available from 2021 Apr 16: https://lexikon.stangl.eu/593/resilienz.

[34] Campbell-Sills L, Cohan SL, Stein MB. Relationship of resilience to personality, coping, and psychiatric symptoms in young adults. Behav Res Ther. 2006;44(4):585–599. 15998508 10.1016/j.brat.2005.05.001

[35] Kunzler AM, Gilan DA, Kalisch R, et al. Aktuelle Konzepte der Resilienzforschung. Nervenarzt. 2018;89(7):747–753. 29796896 10.1007/s00115-018-0529-x

[36] Altmann T Empathie: Markus Antonius Wirtz; 2021 [updated 2021-02-10; cited 2021-02-10; cited 2021 Apr 16]. Available from: https://dorsch.hogrefe.com/stichwort/empathie.

[37] Ekman E, Krasner M. Empathy in medicine: neuroscience, education and challenges. Med Teach. 2017;39(2):164–173. 27934554 10.1080/0142159X.2016.1248925

[38] Shanafelt TD, West C, Zhao X, et al. Relationship between increased personal well-being and enhanced empathy among internal medicine residents. J Gen Intern Med. 2005;20(7):559–564. 16050855 10.1111/j.1525-1497.2005.0108.x PMC1490167

[39] Thomas MR, Dyrbye LN, Huntington JL, et al. How do distress and well-being relate to medical student empathy? A multicenter study. J Gen Intern Med. 2007;22(2):177–183. 17356983 10.1007/s11606-006-0039-6 PMC1824738

[40] Zenasni F, Boujut E, Woerner A, et al. Burnout and empathy in primary care: three hypotheses. Br J Gen Pract. 2012;62(600):346–347. 22781970 10.3399/bjgp12X652193 PMC3381244

[41] Wilkinson H, Whittington R, Perry L, et al. Examining the relationship between burnout and empathy in healthcare professionals: a systematic review. Burn Res. 2017;6:18–29. 28868237 10.1016/j.burn.2017.06.003 PMC5534210

[42] Yuguero O, Ramon Marsal J, Esquerda M, et al. Association between low empathy and high burnout among primary care physicians and nurses in Lleida, Spain. Eur J Gen Pract. 2017;23(1):4–10. 27723375 10.1080/13814788.2016.1233173 PMC5774288

[43] Wündrich M, Schwartz C, Feige B, et al. Empathy training in medical students - a randomized controlled trial. Med Teach. 2017;39(10):1096–1098. 28749198 10.1080/0142159X.2017.1355451

[44] Stangl WC, Stangl W, Wien LF; 2021 cited 2021 Apr 27]. Available from 2021 Apr 27: https://lexikon.stangl.eu/36/coping.

[45] Lemaire JB, Wallace JE. Not all coping strategies are created equal: a mixed methods study exploring physicians’ self reported coping strategies. BMC Health Serv Res. 2010;10(1):208. 20630091 10.1186/1472-6963-10-208 PMC2914035

[46] Boekaerts M. Self-regulated learning: where we are today. Int J Educ Res. 1999;31(6):445–457.

[47] Brady KJS, Trockel MT, Khan CT, et al. What do we mean by physician wellness? A systematic review of its definition and measurement. Acad Psychiatry. 2018;42(1):94–108. 28913621 10.1007/s40596-017-0781-6

[48] Deutsche Gesellschaft für Psychiatrie PuND. Positionspapier der Deutschen Gesellschaft für Psychiatrie, Psychotherapie und Nerven- heilkunde (DGPPN) zum Thema Burnout. Die Psychiatrie. 2012;09(2):19–26.

[49] Kabat-Zinn J. Wherever you go, there you are: mindfulness meditation in everyday life. New York: Hachette Books; 2009.

[50] Kriakous SA, Elliott KA, Lamers C, et al. The effectiveness of mindfulness-based stress reduction on the psychological functioning of healthcare professionals: a Systematic Review. Mindfulness. 2021;12(1):1–28. 32989406 10.1007/s12671-020-01500-9 PMC7511255

[51] Shapiro SL, Schwartz GE, Bonner G. Effects of mindfulness-based stress reduction on medical and premedical students. J Behav Med. 1998;21(6):581–599. 9891256 10.1023/a:1018700829825

[52] Neff K. Self-compassion: an alternative conceptualization of a healthy attitude toward oneself. Self and Identity. 2003;2(2):85–101.

[53] Crosskey L, Curry J. Self-Compassion: conceptualizations, correlates, & interventions. Rev General Psychol. 2011;15(4):289–303.

[54] Neff KD, Germer CK. A pilot study and randomized controlled trial of the mindful self-compassion program. J Clin Psychol. 2013;69(1):28–44. 23070875 10.1002/jclp.21923

[55] Mills A, Gilbert P, Bellew R, et al. Paranoid beliefs and self-criticism in students. Clin Psychol Psychother. 2007;14(5):358–364.

[56] Crosskey L, Curry J. The relationship of clergy burnout to self-compassion and other personality dimensions. Pastoral Psychol. 2011;61:149–163.

[57] Moher D, Liberati A, Tetzlaff J, et al. Preferred reporting items for systematic reviews and meta-analyses: the PRISMA statement. PLoS Med. 2009;6(7):e1000097–e. 19621072 10.1371/journal.pmed.1000097 PMC2707599

[58] Jadad AR, Moore RA, Carroll D, et al. Assessing the quality of reports of randomized clinical trials: is blinding necessary? Control Clin Trials. 1996;17(1):1–12. 8721797 10.1016/0197-2456(95)00134-4

[59] Nicolas Rüsch MD, Sara EE-L Ph.D., Claire Henderson MD Ph.D., et al. Knowledge and attitudes as predictors of intentions to seek help for and disclose a mental illness. Psychiatric Serv. 2011;62(6):675–678. 10.1176/ps.62.6.pss6206_0675 21632739

[60] Kurki M, Sonja G, Kaisa M, et al. Digital mental health literacy-program for the first-year medical students’ wellbeing: a one group quasi-experimental study. BMC Med Educ. 2021;21(1). 10.1186/s12909-021-02990-4 PMC8571980 34742258

[61] Gilham C, Austen E, Wei Y, et al. Improving mental health literacy in post-secondary students: field testing the feasibility and potential outcomes of a peer-led approach. Can J Community Mental Health. 2018;37(1):1–12.

[62] NCT05011955, Villalon Lopez FJ. Mindfulness and intercare based intervention for medicine students (MIIM). https://clinicaltrialsgov/show/NCT05011955. 2021. Accessed 21 01 2022.

[63] NCT04690452, Rojas B. Evaluation of the efficacy and mechanisms of change of compassion cultivation training in medical students. https://clinicaltrialsgov/ct2/show/NCT04690452. 2020. Accessed 21 01 2022.

[64] Kemper KJ, Khirallah M. Acute effects of online mind–body skills training on resilience, mindfulness, and empathy. J Evid Based Complementary Altern Med. 2015;20(4):247–253. 25783980 10.1177/2156587215575816

[65] Kemper KJ, Lynn J, Mahan JD. What is the impact of online training in mind–body skills? J Evid Based Complementary Altern Med. 2015;20(4):275–282. 25962637 10.1177/2156587215580882

[66] Moore S, Barbour R, Ngo H, et al. Determining the feasibility and effectiveness of brief online mindfulness training for rural medical students: a pilot study. BMC Med Educ. 2020;20(1):104. 32252750 10.1186/s12909-020-02015-6 PMC7137339

[67] Danilewitz M, Koszycki D, Maclean H, et al. Feasibility and effectiveness of an online mindfulness meditation program for medical students. Can Med Educ J. 2018;9(4):e15–e25. 30498540 PMC6260511

[68] Lattie EG, Duffecy JL, Mohr DC, et al. Development and evaluation of an online mental health program for medical students. Acad Psychiatry. 2017;41(5):642–645. 28536989 10.1007/s40596-017-0726-0 PMC6367922

[69] Lattie EG, Kashima K, Duffecy JL. An open trial of internet-based cognitive behavioral therapy for first year medical students. Internet Interv. 2019;18:100279. 31534911 10.1016/j.invent.2019.100279 PMC6743024

[70] George DR, Dellasega C, Whitehead MM, et al. Facebook-based stress management resources for first-year medical students: a multi-method evaluation. Comput Hum Behav. 2013;29(3):559–562.

[71] Folkman S, Lazarus RS, Gruen RJ, et al. Appraisal, coping, health status, and psychological symptoms. J Pers Soc Psychol. 1986;50(3):571–579. 3701593 10.1037//0022-3514.50.3.571

[72] Rastegar Kazerooni A, Amini M, Tabari P, et al. Peer mentoring for medical students during the COVID-19 pandemic via a social media platform. Med Educ. 2020;54(8):762–763. 32353893 10.1111/medu.14206 PMC7267157

[73] Schindler A-K, Polujanski S, Rotthoff T. A longitudinal investigation of mental health, perceived learning environment and burdens in a cohort of first-year German medical students’ before and during the COVID-19 ‘new normal’. BMC Med Educ. 2021;21(1):21. 34340659 10.1186/s12909-021-02798-2 PMC8327055

